# Growth Increase in the Herbaceous Plant *Centella asiatica* by the Plant Growth-Promoting Rhizobacteria *Priestia megaterium* HyangYak-01

**DOI:** 10.3390/plants12132398

**Published:** 2023-06-21

**Authors:** HyungWoo Jo, Kyeongmo Lim, Jerald Conrad Ibal, Min-Chul Kim, Hye-Been Kim, Chaeyun Baek, Young Mok Heo, Haeun Lee, Seunghyun Kang, Dong-Geol Lee, Jae-Ho Shin

**Affiliations:** 1 COSMAX BTI, R&I Center, Seongnam 13486, Republic of Korea; chohw@cosmax.com (H.J.); kimhb@cosmax.com (H.-B.K.); cybaek@cosmax.com (C.B.); ymheo@cosmax.com (Y.M.H.); haeun.lee@cosmax.com (H.L.); shyunk@cosmax.com (S.K.); 2 Department of Microbiology, Dankook University, Cheonan 31116, Republic of Korea; 3 Department of Applied Biosciences, Kyungpook National University, Daehak-ro 80, Daegu 41566, Republic of Korea; lkm3519@knu.ac.kr; 4 NGS Core Facility, Kyungpook National University, Daehak-ro 80, Daegu 41566, Republic of Korea; jerald.ibal@gmail.com (J.C.I.); sksalscjf12@naver.com (M.-C.K.); 5 Department of Integrative Biotechnology, Kyungpook National University, Daehak-ro 80, Daegu 41566, Republic of Korea

**Keywords:** biofertilizer, *Centella asiatica*, microbiome, plant growth-promoting activity, *Priestia megaterium*

## Abstract

*Centella asiatica* is a traditional herbaceous plant with numerous beneficial effects, widely known for its medicinal and cosmetic applications. Maximizing its growth can lead to beneficial effects, by focusing on the use of its active compounds. The use of plant growth-promoting rhizobacteria (PGPR) is known to be an alternative to chemical fertilizers. In this study, we used the PGPR *Priestia megaterium* HY-01 to increase the yield of *C. asiatica*. In vitro assays showed that HY-01 exhibited plant growth-promoting activities (IAA production, denitrification, phosphate solubilization, and urease activity). Genomic analyses also showed that the strain has plant growth-promoting-related genes that corroborate with the different PGP activities found in the assays. This strain was subsequently used in field experiments to test its effectiveness on the growth of *C. asiatica*. After four months of application, leaf and root samples were collected to measure the plant growth rate. Moreover, we checked the rhizosphere microbiome between the treated and non-treated plots. Our results suggest that treatment with Hyang-yak-01 not only improved the growth of *C. asiatica* (leaf length, leaf weight, leaf width, root length, root width, and chlorophyll content) but also influenced the rhizosphere microbiome. Biodiversity was higher in the treated group, and the bacterial composition was also different from the control group.

## 1. Introduction

With the ever-increasing population, pressure on arable land to increase crop yield leads to the indiscriminate use of chemical fertilizers, insecticides, pesticides, etc., by farmers. Agrochemical runoff from such land adversely affects life on Earth with bioaccumulation and biomagnification throughout the food chain. Moreover, pesticides utilized to fight against plant illnesses adversely affect beneficial natural insects, soil fertility, and soil microbiota [1], which also impacts human well-being [2].

Plant biotechnology has been tapped for further genetic improvements in crops using conventional plant breeding as well as breeding supplemented with new technology that came from advances in plant and molecular cellular biology. The increased progress of sequencing technology in recent years has made it possible to obtain a large amount of genome information from a variety of plant species. In a review by Zhu et al. [3], the authors mention increasing the yield and improving the quality and herbicide resistance of crops using precise genome editing based on CRISPR-Cas. In addition, new plant breeding technologies (NPBTs), which include genetically modified organisms (GMOs) and gene-edited crops, may possibly play an important role in increasing the quality and quantity of crop yield [4,5]. Nonetheless, the use of these NBPTs and GMOs is still not widely accepted.

One of the challenges that should be considered in agriculture is the development of environmentally wide-ranging and sustainable crop production methods. In recent years, researchers have also considered the potential use of biofertilizers as an alternative for increasing both the yield and quality of crops. Plant growth-promoting rhizobacteria (PGPR) are soil bacteria that colonize the rhizosphere of plants and thrive in the plant rhizosphere and bulk soil. These PGPRs may have direct effects (phytohormone production, improvement of nutrient availability) and indirect effects (enhancement of symbiotic relations, protection from pathogens) that enhance plant growth, and they can be used to alleviate environmental stress and support the nutrition of host plants, thus creating a powerful tool for sustainable agriculture [6].

A wide variety of PGPR strains help to increase crop yields, exhibit biocontrol, and enhance resistance to pathogens [7]. Due to their occurrence in agricultural fields and their activity that promotes plant health in a variety of ways, multiple species of *Bacillus* and *Paenibacillus* are among the most studied [8,9]. A previous study by Lim and Kim [10] investigated the synergistic plant-promoting ability of red pepper and tomato using the PGPR *Bacillus subtilis* AH18 and *Bacillus licheniformis* K11. Both strains produced auxin, exhibited antifungal β-glucanase, and were able to solubilize phosphates, which resulted in an increase in the yield of the above-mentioned host plants. Another study by Probanza and colleagues [11] made use of *Bacillus licheniformis* CECT 5106 and *Bacillus pumilus* CECT 5105 to enhance the growth of *Pinus pinea* plants.

*Centella asiatica* is a nutritionally important plant and a valued traditional medicine in Southeast Asia. It is commonly used as a green leafy vegetable in traditional societies around the world due to its well-known health benefits, which stem from the presence of high amounts of medicinally important triterpenoids and beneficial carotenoids in *C. asiatica* [12,13]. In recent years, it has also gained interest in the field of cosmetology because its extract is a rich source of natural bioactive substances, including triterpenoid saponins, flavonoids, phenolic acids, triterpenic steroids, amino acids, and sugars. These active compounds are effective in improving the treatment of small wounds, hypertrophic wounds, burns, psoriasis, and scleroderma [14]. Among the various chemicals, the most investigated are asiaticoside, madecasosside, asiatic acid, and madecassic acid, due to their dermatological and pharmacological activity [15].

In this study, the potential of the isolated bacteria *Priestia megaterium* (formerly *Bacillus megaterium*) HyangYak-01 as plant growth-promoting treatment was assessed using genomic and in vitro bioassays. This study also demonstrated how its application increased the yield and altered the rhizosphere microbiome of *C. asiatica,* a known medicinal plant, using 16S rRNA amplicon sequences.

## 2. Results

### 2.1. PGPR Activity Results

**Figure 1:**
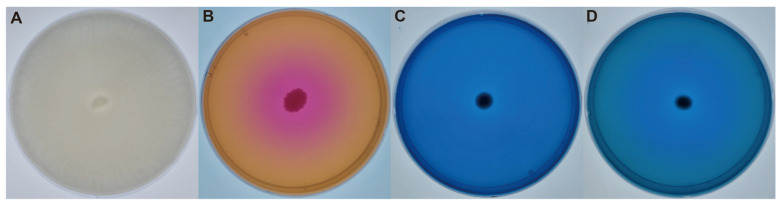
PGPR activity media assay results. (**A**) Phosphate solubilization, (**B**) urease, (**C**) nitrite denitrification, and (**D**) nitrate denitrification were evaluated. All assays showed positive results according to the references.

Various qualitative in vitro assays were performed to screen other PGP traits of HY-01. First, the strain was cultured on an LB agar plate at 30 °C until single colonies were observed. Afterward, the single colonies were inoculated into PGP trait assay plates using the toothpick method. IAA-producing activity was evaluated using the Salkowski reagent method to ensure that HY-01 had this trait even if it was screened for IAA-producing activity (Figure S1). For the phosphate solubility assay containing tricalcium phosphate (TCP), the appearance of a halo zone around the inoculated strain indicated the inorganic phosphate solubilization ability of HY-01 and a positive result. For the urease test, agar plates were supplemented with phenol red, calcium acetate, and urea. The color change from yellow to bright pink indicated a pH change caused by the ammonia release from ureolytic activity. For the denitrification assay targeting both nitrate and nitrite, the strain formed a blue-colored colony, which was considered a positive result (Figure 1A–D). Other PGP traits, such as siderophore and cellulase activity, were also tested and showed negative results.

### 2.2. Field Test

**Figure 2:**
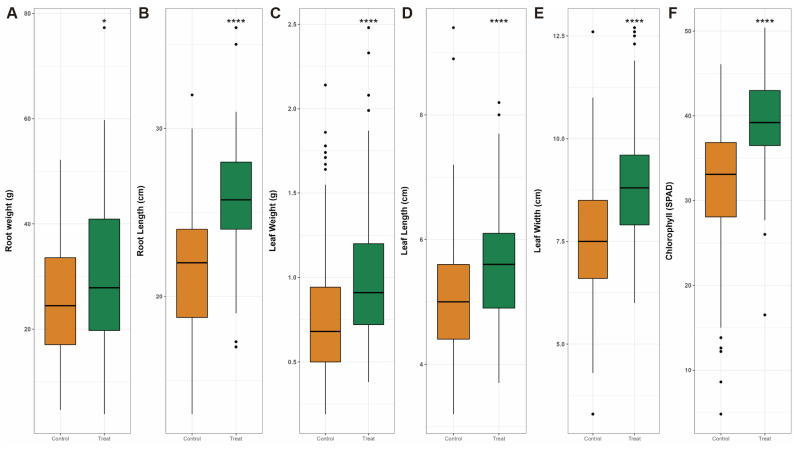
Comparative analysis of *C. asiatica* growth values between the control and treated groups of *C. asiatica*. (**A**) Root weight, (**B**) root length, (**C**) leaf weight, (**D**) leaf length, (**E**) leaf width, and (**F**) chlorophyll content. A Student’s *t*-test was used to calculate statistical differences; * *p* ≤ 0.05, **** *p* ≤ 0.0001.

Treated and non-treated (control) groups of HY-01 were observed. For the treated group, HY-01 cells were first cultured and counted and then allowed to reach 10^9^ CFU/mL. After dilution with sterilized water to 1% of the original culture, the solution was treated directly on a *C. asiatica* plant once every week. Meanwhile, for the non-treated group, distilled water without the strain of interest was used for spraying. *C. asiatica* plants were grown in the field for four months, and the treatment started one week after transplanting. After harvesting, leaf and root samples were collected from both the treated and control groups. Six different growth parameters, including root weight, root length, leaf weight, leaf length, leaf width, and chlorophyll content, were measured from the collected leaf and root samples for the PGP-treated and control groups of *C. asiatica* to conduct a phenotypical comparative analysis. The results showed a significant difference between the two groups (Figure 2). In all values, the treated group showed higher values than the control group, indicating that treatment with the strain HyangYak-01 increased the growth rate of the planted *C. asiatica*.

### 2.3. Soil Physicochemical Property Analysis

The physicochemical properties of the soil where *C. asiatica* was planted were analyzed to evaluate the effect of HY-01 application on soil properties. The results showed significant differences between the treated and untreated soil (Table 1). Soil organic matter (SOM), pH, available phosphorus, and extractable cations (K, Ca, and Mg) were measured. The treated soil showed lower values for pH, SOM, available phosphorus, and extractable K cation, while extractable Ca and Mg were higher compared to the untreated soil.

### 2.4. Whole-Genome Analysis

**Figure 3:**
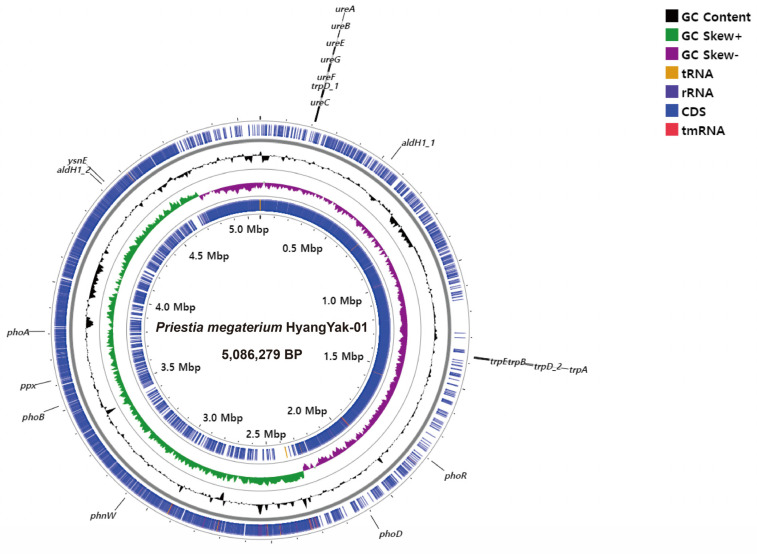
Circular representation of *P. megaterium* HyangYak-01 using Proksee. The scale is shown in mega-base pairs at the central circle. PGP-related genes were highlighted at the outermost circle.

The whole genome of HY-01 was sequenced and analyzed to further confirm its PGP traits using long- and short-read sequences obtained from hybrid sequencing using MGI and Nanopore. After the assembly and polishing steps, the complete genome was obtained and subsequently annotated for functional gene analysis. The general features of the completed genome are provided below (Table 2). As a result of the annotation, genes related to PGP traits were manually searched and identified, which are summarized in Figure 3 and Table 3.

### 2.5. Rhizosphere Microbiome Analysis

**Figure 4:**
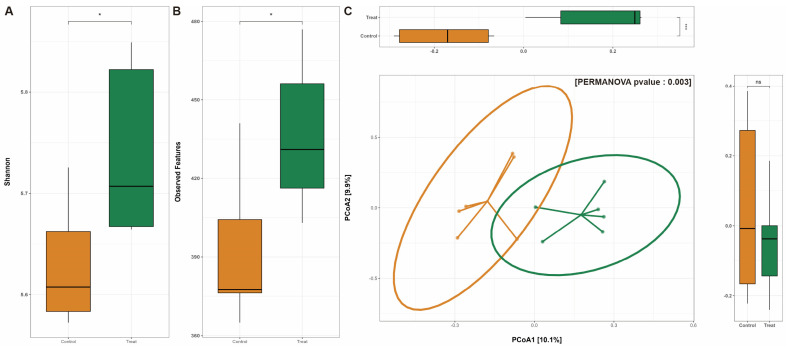
Comparison of *Centella asiatica* rhizosphere microbiome diversity. (**A**) The Shannon index and (**B**) observed features were chosen for the alpha diversity index. (**C**) The principal coordinates analysis (PCoA) was calculated with the Bray–Curtis dissimilarity; * *p* ≤ 0.05, *** *p* ≤ 0.001, ns for no significant.

To investigate the shift in *C. asiatica* rhizosphere microbiota due to PGP treatment, we measured the bacterial community composition and biodiversity. In terms of alpha diversity, the PGP-treated group showed higher values than the control group using two indices, i.e., the observed features and Shannon index (Figure 4A,B), which indicates that the PGP-treated group’s rhizosphere microbiota was more diverse compared to the control group. A difference was also observed in beta diversity. Beta diversity was measured and compared using the Bray–Curtis dissimilarity. The result showed a separate tendency between the two groups with a certain percentage of the two axes in the PCoA plot (Figure 4C). Afterward, we analyzed the bacterial relative abundance in each community to confirm which taxa were contributing to such a difference in biodiversity.

**Figure 5:**
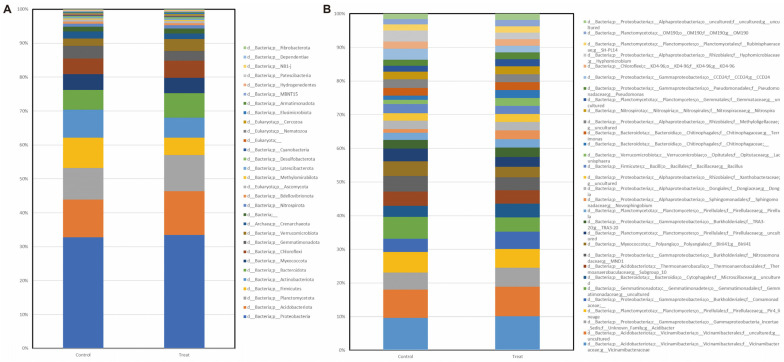
Relative abundance of *C. asiatica* rhizosphere microbiome at the (**A**) phylum and (**B**) genus levels. The top 30 taxa are sorted by abundance.

Abundance was measured at the phylum level. Compared to the control group, in the PGP-treated group, the abundance of Actinobacteriota and Firmicutes was decreased and Bacteroidota was slightly increased, but there was no statistical difference (Figure 5).

## 3. Discussion

*C. asiatica* is an important herb that has been widely used in the Orient for hundreds of years and has recently gained high interest in the West due to its medicinal properties, such as anti-inflammatory, antisporiatic, antiulcer, hepatoprotective, anticonvulsant, and sedative effects, among others [16]. Additionally, it is also being explored for its potential use in cosmetics. Maquart et al. [17] conducted cell culture studies on a metabolite from *C. asiatica*, asiatic acid, which was found to be responsible for collagen synthesis in human fibroblasts. Moreover, asiatic acid, madecassic acid, asiaticoside, and madecassoside were also found to stimulate collagen synthesis [18]. Given its ability to stimulate collagen, *C. asiatica* has been used in skin care products to restore skin firmness and improve skin appearance [19]. In this study, we aimed to increase the yield of *C. asiatica* using a potential plant growth-promoting rhizobacterium (PGPR), HY-01, and analyzed its effect not only on the growth of *C. asiatica* but also on the rhizosphere microbiome.

*Priestia megaterium* is a PGPR that was previously known as *Bacillus megaterium*. Over the years, this Gram-positive bacterium has been found to tolerate different concentrations of sodium chloride and produce plant auxin [20]. Strain BPR2, which was previously known as *Bacillus megaterium* BPR2 but is now identified as *Priestia megaterium* BPR2, was isolated from the root tissues of a salt marsh halophyte. Priestia is a genus of mostly Gram-positive, rod-shaped bacteria in the family Bacillaceae from the order Bacillales, with the species type being *Priestia megaterium* [21]. Strain HY-01, which was used as the main agent in this research, was isolated from the rhizosphere soil of *Centella asiatica*. This strain was selected for its outstanding auxin-producing activity among the candidates (Figure S1).

We analyzed the PGP properties of strain HY-01 using in vitro assays to demonstrate its ability to produce IAA, solubilize phosphate, produce urease, and denitrify nitrate (Figure 1). Additionally, we performed whole genome analysis and searched for genes involved in the PGP properties exhibited by HY-01. To test for IAA production, we conducted a Salkowski’s test and observed a pink color, indicating that HY-01 is capable of producing this phytohormone involved in plant growth and development, including cell elongation, cell division, tissue differentiation, and apical dominance [22]. We identified genes involved in tryptophan and IAA synthesis in the genomic analysis (*aldH*, *trpE*, *trpD*, *trpA*, *trpB*, and *ysnE*). These genes play a role in the tryptophan-dependent pathways of bacterial indole-3-acetic acid [23]. In terms of phenotypic data, the treated group showed an increase in root length and biomass compared to the control group, indicating that the use of strain HY-01 contributed to the growth and yield of *C. asiatica*. The strain also exhibited urease activity, with genes involved in urease activity identified as *ureA*, *ureB*, *ureC*, *ureE*, *ureF*, and *ureG*. *ureA*, *ureB*, and *ureC* genes are three different structural genes of the urease operon [24], while *ureE*, *ureF*, and *ureG* genes are required for the assembly of the nickel metallocenter, which contribute to urease active sites [25]. Urease converts urea to ammonia and carbon dioxide, providing nitrogen directly to the plant in the rhizosphere [26,27]. The strain was also able to solubilize phosphate, with genes involved in phosphate solubilization identified as *phnW*, *phoA*, *phoB*, *phoD*, *phoR*, and *ppx*. For phosphate solubilization activity, the related genes are mostly part of the phosphorus cycle. *phoA*, *phn*, and *ppx* genes are involved in mineralization. *phoB* and *phoR* genes are phosphorus absorption-regulating genes, and *phoD* encodes alkaline phosphatase. Lastly, the *phnW* gene is related to aminotransferase [28,29]. Phosphorus is an essential macronutrient for biological growth and development, and microbes must convert it into a soluble form for plant uptake. In addition to the traits tested, several other PGP traits are considered important features to assess HY-01′s potential as PGPR.

A field experiment was conducted in appropriate soil conditions. According to Devkota et al. [30], *C. asiatica* showed the highest growth rate in soil composed of around 20% to 40% of sand, and since it is a tropical plant, water-holding capacity is important. The field soil was silty soil, and thus appropriate for the growth of *C. asiatica.* Soil physicochemical properties showed different results between the HY-01-treated and non-treated groups (Table 1). Soil organic matter (SOM) is a vital factor for crop productivity due to its various effects on soil properties [31]. Our results showed decreased SOM content in the treated group. Microorganisms capable of degrading fiber, such as *Bacillus megaterium*, are known to affect the degradation of organic nutrients [32]. From this, it can be suggested that SOM contained in HY-01-treated soil was degraded more and taken up by the plants compared to the untreated group. In the case of soil pH, the HY-01-treated soil showed significantly lower pH than the untreated soil. According to previous research, the application of PGPR strains decreased soil pH supposedly due to the production of organic acids [33]. Additionally, decreased pH affected available P and Ca^2+^ ions in the soil. In general, phosphorus availability is highest around pH 6.5, and it is fixed by calcium at higher pH [34]. Our results showed a lower amount of P and a higher amount of Ca^2+^ ions in the treated soil than in the untreated soil. Considering this result with soil pH, a lower amount of available phosphorus in the treated soil indicates that more phosphorus was solubilized in the treated soil since the average soil pH was 6.92, and subsequently, higher amounts of it were used by plants. A higher amount of exchangeable Ca^2+^ ions in the treated soil also suggested that a lower amount of phosphorus was fixed. In the case of the K^+^ and Mg^2+^ ions, the results showed a higher amount of Mg^2+^ and a lower amount of K^+^ in the treated than untreated soil. Numerous studies have reported the antagonism between K^+^ and Mg^2+^ ions in the soil. Due to the absence of a specific uptake system, K^+^ and Mg^2+^ ions are competitively absorbed by plant roots [35]. The field experiment results (Figure 2) showed a significantly higher growth rate in the treated group, and symptoms of nutrient deficiency were not observed for either *C. asiatica* group after sampling. Considering this phenotypic result, the difference in extractable K^+^ and Mg^2+^ levels in treated soil suggested that the K^+^ ion demand was higher in the treated soil due to its growth rate, and, therefore, Mg^2+^ ion uptake was inhibited.

We also examined the changes in the rhizosphere microbiome following the application of HY-01 and compared it with a non-treated control group in a field experiment. We obtained a total of 251,368 reads from sequencing, and after pre-processing, denoising, and feature extraction, we obtained 6684 ASVs. Upon analyzing the alpha and beta diversity, our data revealed that there was an increase in alpha diversity (Shannon index and observed species) in the treated group compared to the control. While plant species tend to have distinct rhizobial communities, our results showed that alpha diversity was significantly higher in the treated group. Previous studies have shown a similar trend, where healthy greenhouse tomato RS samples had greater bacterial diversity than diseased RS samples [36]. Additionally, a study by Filion et al. [37] demonstrated that healthy seedlings of *Picea mariana* have higher diversity compared to diseased seedlings. These findings suggest that increased bacterial diversity indicates a healthier plant. For beta diversity, which measures differences between samples, we utilized the Bray–Curtis dissimilarity. Our results indicated that there was clustering between the two groups (treated and control), and they were statistically different using Permanova (999 permutations, *p* < 0.003). Although there were no significant changes detected across the taxonomic composition when using LefSE, we also examined the genera and compared changes in the two treatments. Zooming in on the top 30 genera, we found an increase in g_Vicinamibacteraceae, g_Acidibacter, f_Comamonadaceae; g_Novosphingobium, g_Lacunisphaera, f_Chitinophagaceae; g_Nitrospira, and g_Pseudomonas in the treated group. Mannaa et al. [38] showed that acidibacter plays ecological and plant growth-promoting roles in pine trees while also thriving in soils at low pH [39], which coincides with the pH results on the physicochemical properties. Meanwhile, a study by Wen et al. [40] found that an increased abundance of comamonadaceae played a role in suppressing *Fusarium oxysporum* in the rhizosphere of cucumber plants. A similar result was obtained in a study by Dai et al. [41], where they found that inoculation of *Lysobacter antibioticus* 13-6 enhanced maize yield and caused changes in its soil rhizosphere communities with increased abundance for *Nitrospira*. The decrease in soil organic matter may also be attributed to the increase in Chitinopagaceae and Novosphingobium, which are known degraders of organic compounds [42]. Although we found a decline in the abundance of *Bacillus* in the treated soil compared with the control, it can be suggested that the treatment had both direct and indirect effects on the growth of *C. asiatica*. The direct effects may have been due to the increased production of IAA and phosphate solubilization based on the qualitative and genomic analysis, while the indirect effect could have been induced by the shift in rhizosphere microbiome, in which there was an increase in genera that have been reported to exhibit plant-promoting properties. A limitation of this study is that we were unable to track HY-01 (*Priestia megaterium*) after application. However, we assume that the weekly treatment for four months would have been sufficient for the strain to be able to promote plant growth and shift the rhizosphere microbiome.

Taken together, the in vitro assays and genomic analyses provided clear evidence that strain HY-01 exhibited plant growth-promoting properties. The phenotypic data, including increased yield and biomass (root length, root weight, leaf length, and leaf weight) of the treated *C. asiatica* plants in comparison with the control group supported our hypothesis that HY-01 would be a viable biofertilizer for cultivating *C. asiatica*. Moreover, the changes in soil physicochemical properties, increased diversity, and abundance of plant growth-promoting genera in the rhizosphere of the treated samples could explain indirect effects on promoting plant growth. Based on the comprehensive results obtained, it is expected that the HY-01 strain can be used in environmentally friendly farming methods with reduced use of chemical pesticides, and the produced *C. asiatica* can be utilized as a raw material for improving skin conditions in various industries, including the cosmetics industry.

## 4. Materials and Methods

### 4.1. Rhizobacteria Isolation

Strain HY-01 was isolated from the rhizosphere soil of CA. The soil was diluted with 0.85% NaCl solution, spread on R2A agar media, and subsequently incubated at 30 °C for 48 h. Isolated bacteria were screened using the IAA test to obtain a number of candidates. *Priestia megaterium* HyangYak-01 was selected not only for its outstanding IAA-producing activity compared to the other candidates but also because *Priestia megaterium was* previously known as *Bacillus megaterium,* which is a well-known PGPR.

### 4.2. Plant Growth-Promoting Activity Test

#### 4.2.1. Indole-3-Acetic Acid

The Salkowski reagent method was used to evaluate IAA-producing activity. A Salkowski reagent was prepared with 0.5M ferric chloride and 35% perchloric acid. HY-01 was inoculated in LB broth with 0.3% of L-tryptophan and incubated at 30 °C for 24 h. After incubation, 1 mL of the cultured broth was centrifuged, and 50 μL of the supernatant was mixed with 1 mL of the Salkowski reagent. The mixed solution was subsequently reacted in dark conditions for 30 min, and the activity was confirmed with the colorimetric method [43].

#### 4.2.2. Phosphate Solubilization

The phosphate solubilization activity was tested using an agar-plated assay. First, 5 g of Ca3(PO_4_)_2_, 10 g of sucrose, 0.5 g of yeast extract, 0.27 g of NH_4_NO_3_, 0.2 g of KCl, 0.1 g of MgSO_4_·7H_2_O, 0.001 g of MnSO_4_, and 0.001 g of FeSO_4_·7H_2_O were dissolved in 1 L of dH_2_O, and then the pH was adjusted to 7.0. The solution was sterilized at 121 °C for 15 min. The sterilized medium was poured into a petri dish where HY-01 was inoculated and incubated for 14 days at 30 °C [44].

#### 4.2.3. Urease Activity

A urease activity assay was conducted using a urea agar plate assay. The medium was composed of 20 g of urea, 1 g of dextrose, 1 g of peptone, 5 g of NaCl, 2 g of KH_2_PO_4_, 122 mg of phenol, and 15 g of agar powder dissolved in 1 L of distilled water. A single colony was placed in the middle of the plate. The plates were incubated for five days at 30 °C [45].

#### 4.2.4. Denitrification

Denitrification activity was assessed using a plate assay. The medium was prepared separately with solutions A and B. Solution A was composed of 1 g of NaNO_2_ (or NaNO_3_), 1 g of asparagin, and 5 mL of 1% bromothymol blue solution in 50% ethanol dissolved in 500 mL of distilled water. Solution B was composed of 8.5 g of sodium citrate, 1 g of MgSO_4_·7H_2_O, 0.05 g of FeCl_3_·6H_2_O, 1 g of KH_2_PO_4_, and 0.2 g of CaCl_2_·2H_2_O dissolved in 500 mL of distilled water. Solution A and B were subsequently mixed, and the pH was adjusted to 7.0. The plates were incubated for 3 days at 30 °C [46].

### 4.3. Treatment and Plant Sample Collection of Centella asiatica

A field treatment experiment was carried out at Cosmax HyangYak herb garden, located in 11-37, Yugugyebong-gil, Yugu-eup, Gongju-si, Chungcheongnam-do, Korea (latitude: 126.97°, longitude: 36.61°). The experiment was conducted in two separate beds, one for the PGP-treated group and another for the control group. For each group, 100 of *C. asiatica* was planted, which is statistically sufficient to represent the population. Strain HyangYak-01 was cultivated in a shaking incubator for 48 h at 30 °C and 150 RPM until it reached 10^9^ CFU/mL. Eventually, it was diluted in sterilized water to 1% of the original culture and finally sprayed on 14 m^2^ of *C. asiatica* grown in the field once every week. *C. asiatica* samples were harvested after four months of growth (27 April–12 August 2022). Afterward, we collected leaf and root samples from both treated and non-treated (control) *C. asiatica* groups to compare growth values, leaf width, leaf length, leaf width, root length, root weight, and chlorophyll content. Chlorophyll content was measured using a chlorophyll meter. In addition, 10 g of rhizosphere soil samples were collected randomly from six different spots in both the treated and control plots, and 1 kg of soil was also collected from the treated and control plots to check soil physicochemical properties.

### 4.4. Soil Physicochemical Property Analysis

The soil samples used for the physicochemical property analysis were dried at RT for 3 days and then sieved with a 2 mm pore size. Soil organic matter (SOM), pH, available phosphorus, and extractable cations (K, Ca, and Mg) were measured according to the analysis method of the National Institute of Agricultural Science. Soil pH was measured from a soil solution. SOM was measured with the Walkley–Black method, with measured absorbance at 610 nm wavelength, and available phosphorus was measured with the molybdenum blue method, conducted with colorimetric quantification at 660 nm wavelength. For both SOM and available phosphorus, UV-2401PC (Shimadzu Corporation, Kyoto, Japan) was used. In the case of extractable cations, the ions were extracted with 1N NH_4_OAc (pH 7.0) and analyzed with Integra XL ICP-OES (GBC, Melbourne, Australia).

### 4.5. DNA Extraction, Library Preparation, and Sequencing

#### 4.5.1. Method for 16S rRNA Amplicon Sequencing

Genomic DNA for metagenome sequencing was extracted from the *C. asiatica* soil rhizosphere. The extraction was conducted with a DNeasy PowerSoil Kit (Qiagen, Hilden, Germany) without any modification. The concentration of extracted DNA was measured with a Qubit fluorometer 2.0 (Waltham, MA, USA). Subsequently, the extracted DNA was used for amplicon sequencing library preparation. The V4 region on the bacterial 16S rRNA gene was used as the targeted site for amplification. A polymerase chain reaction (PCR) was carried out in two steps. First, the initial PCR started with denaturation at 95 °C and moved on to 25 cycles consisting of annealing at 55 °C and extension at 72 °C. Next, the DNA purification step with AMPure XP bead (Beckman, Pasadena, CA, USA) was carried out after each PCR. The quantity and size of the final library were checked with an Agilent 2100 Bioanalyzer (Santa Clara, CA, USA). Sequencing was performed using the Illumina MiSeq platform (Illumina, CA, USA) at the KNU NGS Core Facility, Daegu, South Korea, following the manufacturer’s protocols.

#### 4.5.2. Whole-Genome Sequencing

Genomic DNA of *P. megaterium* HyangYak-01 for whole-genome sequencing was extracted with a Promega Wizard Genomic DNA Purification Kit (Promega, WI, USA) following the provided protocol. The quality and quantity of the extracted DNA were measured with Qubit fluorometer 2.0 and NanoDrop One^C^ Microvolume UV-Vis Spectrophotometer (Thermo Fisher Scientific, MA, USA). The confirmed DNA was subsequently sequenced with Oxford Nanopore MinION Mk1C flatform using flow cell (R10.4.1) and a native barcoding kit 24 V14 (Oxford Nanopore Technologies, Oxford, UK) for long-read sequencing and DNBSEQ-G400RS flatform (MGI tech, Shenzhen, China) with a PE50 kit for short-read sequencing. Sequencing was performed using the Illumina MiSeq platform (Illumina, CA, USA) at the KNU NGS Core Facility, Daegu, Republic of Korea, following the manufacturer’s protocols.

#### 4.5.3. Bioinformatic Analysis

Raw metagenome sequence data were analyzed with the Quantitative Insights into Microbial Ecology2 (Qiime2) analysis pipeline (version 2021.04). The imported raw sequences were filtered with DADA2. Eventually, 340,000 reads were obtained in total. Next, the filtered reads were used for taxonomy information assignments. The SILVA database (SILVA SSU version 138.1) was used as a reference database. The Qiime2 output files including the taxonomy file subsequently imported into R for statistical analysis and visualization using the phyloseq package (version 1.40.0) [47]. The imported data were subsequently analyzed for biodiversity. The observed features and the Shannon index were used to indicate alpha diversity with the Microbiome package (version 1.18.0) [48], and a Student’s t-test was conducted for statistical purposes. Beta diversity was measured with the Bray–Curtis dissimilarity using the vegan package (version 2.62) [49]. Relative abundance was calculated and sorted with the top 30 taxa at the phylum and genus levels.

Both long- and short-read sequences for the whole-genome analysis were used for hybrid assembly with Maryland Super Read Cabog Assembler (MaSuRCA, version 4.1.0). Assembled contigs were subsequently scaffolded with CSAR and polished with Polypolish. The polished genome was confirmed as a complete genome with the NCBI Prokaryotic Genome Annotation Pipeline (PGAP, version 6.5). Functional gene annotation and visualization were conducted with Proksee.

## Figures and Tables

**Table 1:** Soil physicochemical properties of *C. asiatica* planted in the soil.

Group	pH (1:5)	SOM (g kg^−1^)	Avail. P (mg kg^−1^)	Extractable Cation (cmol_c_ kg^−1^)		
				K	Ca	Mg
Control	7.91 ± 0.01	19.45 ± 0.06	524.42 ± 5.45	0.38 ± 0.02	11.01 ± 0.01	3.89 ± 0.08
Treated	6.92 ± 0.02	16.52 ± 0.02	450.2 ± 0.14	0.26 ± 0.01	11.83 ± 0.03	4.16 ± 0.03
Significance	****	****	**	**	***	*

A Student’s *t*-test was conducted to calculate statistical differences; *, *p* ≤ 0.05; **, *p* ≤ 0.01; ***, *p* ≤ 0.001; ****, *p* ≤ 0.0001.

**Table 2:** General features of *P. megaterium* HyangYak-01′s assembled genome.

Features	Values
Genomic size (bp)	5,086,279
GC content (%)	38.2
Total genes	5289
CDSs	5111
Pseudo-genes	48
Ribosomal RNAs	44
Transfer RNAs	126
Other RNAs	8

**Table 3:** PGP-related genes found in the assembled HY-01 genome.

Related PGP Traits	Genes
IAA-producing	*aldH*
	*trpE*
	*trpD*
	*trpA*
	*trpB*
	*ysnE*
Urease activity	*urea*
	*ureB*
	*ureC*
	*ureE*
	*ureF*
	*ureG*
Phosphate solubilization	*phnW*
	*phoA*
	*phoB*
	*phoD*
	*phoR*
	*Ppx*

## Data Availability

All raw sequencing data for the microbiome used in this study were uploaded to the NCBI Sequence Read Archive (SRA) under the accession number PRJNA956735. Whole-genome sequencing data used in this study were uploaded to NCBI under the accession number PRJNA956741.

## Supplementary Materials

The following supporting information can be downloaded at: https://www.mdpi.com/article/10.3390/plants12132398/s1, Figure S1: IAA-producing activity assay with the Salkowski reagent method. (A) Control without cell culture and (B), (C), (D) use HY-01 in triplicate. The result indicated HY-01 has IAA-producing activity.
