# Supplementary figures and images for: Growth Increase in the Herbaceous Plant Centella asiatica by the Plant Growth-Promoting Rhizobacteria Priestia megaterium HyangYak-01
Source: Plants (Basel). 2023 Jun 21;12(13):2398. doi: 10.3390/plants12132398 (PMC10346698; doi:10.3390/plants12132398)

**A**

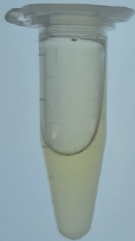

**B**

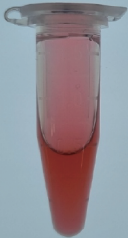

**C**

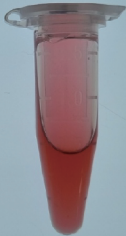

**D**

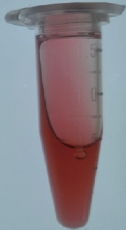

Supplement: Supplementary file 1 [file plants-12-02398-s001.zip › plants-2389522-supplementary.pdf]
